# Challenges of reproducible AI in biomedical data science

**DOI:** 10.1186/s12920-024-02072-6

**Published:** 2025-01-10

**Authors:** Henry Han

**Affiliations:** The Laboratory of Data Science and Artificial Intelligence Innovation, Department of Computer Science, School of Engineering and Computer Science, Baylor University, Waco, TX 76798 USA

**Keywords:** AI, Reproducibility, Biomedical data, Game-theory

## Abstract

Artificial intelligence (AI) is revolutionizing biomedical data science at an unprecedented pace, transforming various aspects of the field with remarkable speed and depth. However, a critical issue remains unclear: how reproducible are the AI models and systems employed in biomedical data science? In this study, we examine the challenges of AI reproducibility by analyzing the factors influenced by data, model, and learning complexities, as well as through a game-theoretical perspective. While adherence to reproducibility standards is essential for the long-term advancement of AI, the conflict between following these standards and aligning with researchers’ personal goals remains a significant hurdle in achieving AI reproducibility.

## Introduction

AI is revolutionizing biomedical and health fields. Advanced algorithms and models now excel at analyzing massive datasets, uncovering patterns and insights imperceptible to human analysts. In genomics, for instance, AI aids in predicting gene functions and understanding genetic predispositions to diseases. In proteomics, AI’s pattern recognition capabilities are pivotal in elucidating protein structures, functions, and interactions. For example, AlphaFold3, developed by DeepMind, has revolutionized protein structure prediction with transformer-based AI, achieving unprecedented accuracy [1]. Awarded the 2024 Nobel Prize in Chemistry, it addresses a decades-long challenge in molecular biology.

AI also plays a transformative role in spatial omics, such as spatial transcriptomics, uncovering spatial cell interactions and gene expression patterns that advance cancer research and personalized medicine [2]. Machine learning models like CNNs and GNNs analyze tumor microenvironments, identify immune infiltration, and predict outcomes, aiding immunotherapy, and drug development. Additionally, AI is accelerating drug discovery by identifying potential candidates, simulating molecular interactions for safer and more effective drugs, and tailoring treatments using diverse data sources like electronic health records (EHRs) and wearable devices. This revolution not only shortens drug development cycles but also enhances therapy success rates and enables precise, personalized healthcare [3].

A key issue that remains unclear is the reproducibility of biomedical AI models and systems. Reproducibility refers to the ability of an AI model or system to repeat an experiment and obtain the same results. For instance, AlphaFold3’s reproducibility allows its protein structure predictions to be independently verified by other researchers. A common misconception is that reproducibility can be achieved simply by making the relevant codebase publicly available, enabling researchers to use the same code to replicate the results. However, it is highly likely that running the open-source code may not yield the same results or performance due to the non-deterministic nature of learning models, variations in software systems, differences in hardware settings, data preprocessing variability, and the use of different optimizers. For example, a deep learning model with batch normalization under Stochastic Gradient Descent (SGD) optimization might generate different results in each run due to the random data variations introduced by batch normalization and SGD [4]. In this study, we explore the challenges of AI reproducibility in biomedical data science, a critical yet underexplored topic with both theoretical and practical significance.

## The sources of biomedical AI irreproducibility

In biomedical data science, despite its critical importance, AI reproducibility has not received as much attention as explainability and other AI ethics. Explainability involves understanding and interpreting the decisions made by AI models, enabling users to comprehend these decisions, which is crucial for establishing trust and transparency [5]. However, without reproducibility — the guarantee that results are consistent and reliable over time, across various datasets, and upon repeated runs — the validity of even the most transparent AI system’s results might be questioned. Discussing transparency and interpretation becomes challenging if an AI model or system cannot consistently reproduce its results. In the biomedical field, where decisions about disease diagnosis, drug development, and personalized patient care are of significant consequence, reproducibility is foundational.

The irreproducibility of AI in biomedical data science often stems from several key factors, including the inherent non-determinism of AI models, data variations, data preprocessing, computational costs, and hardware variations.

### Inherent non-determinism of AI models

Many AI models, particularly ensemble learning methods and certain deep learning architectures like large language models (LLMs), exhibit non-deterministic behavior. This arises from various sources inherent in the models’ architecture, training processes, hardware acceleration, or even mathematical definitions. For instance, LLMs may produce different outputs for the same input due to stochastic sampling during text generation, randomization in training processes such as data shuffling and weight initialization, and hardware-induced variability from parallel computing resources.

Deep learning models, such as convolutional neural networks (CNNs), are particularly prone to non-determinism, even though they can be mathematically deterministic in model design. Factors contributing to this include random weight initialization, mini-batch gradient descent, dropout regularization techniques, and hardware acceleration. These elements can lead to different training runs converging to various local minima on the error surface. The use of non-deterministic optimization methods, such as Stochastic Gradient Descent (SGD) and its variants, which utilize random mini-batches of data, further compounds this effect.

Additionally, architectural decisions like the choice of activation functions and the use of dropout layers for regularization introduce variability that impacts reproducibility. For example, the random deactivation of neurons during training via dropout can lead to different model behaviors across runs. While setting random seeds can mitigate some of these variations, it cannot eliminate them entirely. Activation functions with sharp transitions, like sigmoid or tanh, can amplify floating-point precision issues, particularly when combined with hardware acceleration (e.g., GPUs or TPUs). The ReLU (Rectified Linear Unit) activation function can produce dead neurons during training, where neurons output zero for all inputs and stop contributing to the learning process. Furthermore, hardware acceleration (e.g., using GPUs) itself introduces random data variations due to parallel processing and floating-point precision limitations.

### Data variations

AI systems are highly dependent on the quality and completeness of their training data, which directly affects their performance and reproducibility. Variations between training and testing datasets can lead to irreproducibility issues, such as overfitting. For example, a model trained on high-quality genomic data may perform poorly when tested on datasets containing artifacts.

Incomplete training datasets that lack representation from diverse demographic groups can also result in inadequate performance on underrepresented populations. For instance, a dataset predicting diabetes risk may overrepresent middle-aged urban adults, neglecting younger or rural populations. This imbalance can cause the model to generalize poorly, leading to higher error rates or misdiagnoses for underrepresented groups, such as missing early-onset diabetes in younger individuals.Moreover, this underrepresentation can cause the model’s performance to vary dramatically across different test settings or populations, ultimately undermining its reproducibility.

Additionally, data leakage—where information from the test set inadvertently influences the training process—can artificially inflate performance metrics, causing models to fail on independent datasets, thereby hurting AI reproducibility. Beyond artifacts in data acquisition, data leakage often stems from improper data handling, such as applying normalization or feature selection before splitting data into training and test sets.

### Data preprocessing

Data preprocessing is crucial for the reproducibility of AI models, particularly in biomedical data science. Techniques such as normalization, feature selection, vectorization, and dimensionality reduction and data integration significantly influence training and downstream analysis. The choice of methods within these processes can lead to variations in training and test data quality or introduce randomness into the training process. For example, batch normalization—a regularization technique widely used in deep learning—introduces random data variations primarily during training due to the computation of mini-batch statistics.

As mentioned, improper normalization or feature selection applied before splitting data into training and test sets can result in data leakage, further impacting training. Additionally, dimensionality reduction methods like t-SNE and UMAP are inherently non-deterministic, as they rely on solving non-convex optimization problems with multiple possible solutions, contributing to variability in data preprocessing [5, 6]. These challenges are particularly relevant for large-scale, complex biomedical datasets, where optimal preprocessing methods have yet to be established.

### Computational costs and hardware variations

Computational costs for AI models, particularly in complex biomedical domains, are substantial and significantly impact reproducibility. For example, models like AlphaFold3 tackle NP-hard problems, with computational complexity rising exponentially with input size, making third-party verification resource-intensive. The original AlphaFold required 264 h of training on Tensor Processing Units (TPUs), while optimized versions like FastFold reduced this to 67 h [1]. However, the high computational demands can still deter independent researchers from replicating these results, thereby hindering broader reproducibility efforts.

Additionally, hardware introduces variability in computing. GPU and TPU computations can produce non-deterministic results due to parallel processing, floating-point operations, stochastic rounding, and software differences in frameworks like TensorFlow and PyTorch. These hardware-induced variations, coupled with high computational costs, hinder independent verification efforts, and exacerbate reproducibility challenges.

## Key challenges in achieving reproducible AI in biomedical data science

Achieving reproducible AI in biomedical data science is challenging due to inherent complexities in data, models, and learning processes, compounded by a game-theoretical dilemma. These complexities create multiple sources of irreproducibility that are difficult to address effectively.

### Data complexity

Data complexity refers to the challenges arising from the characteristics and quality of the input data used in biomedical AI models. These challenges include high dimensionality, where datasets with numerous features increase computational demands and complicate modeling, and heterogeneity, which involves variations in data types like text, images, and numerical values. Additionally, multimodality, or the need to combine diverse data sources such as genomic data with imaging or clinical records, further amplifies complexity. Issues like missing data and noise require imputation or cleaning, often introducing variability, while bias and imbalance in datasets can result in models that poorly generalize to underrepresented populations or classes. Managing these complexities is essential to building effective and reproducible AI models. High-dimensional, heterogeneous, and multimodal datasets, coupled with missing or imbalanced data, complicate preprocessing and introduce variability, making it challenging to standardize reproducible pipelines, especially in biomedical fields.

#### Impact of data complexity on preprocessing

Biomedical datasets often contain diverse data types, such as genomic sequences, imaging, and clinical records, each characterized by high dimensionality and heterogeneity. These features make it challenging to design preprocessing techniques that effectively standardize data without introducing inconsistencies. For example, integrating disparate data structures from various sources often leads to conflicts in scaling, alignment, or representation, which can negatively affect reproducibility. These issues underscore the difficulty of ensuring consistent preprocessing for complex datasets.

#### Challenges in multimodal data and missing data

The multimodal nature of biomedical data further compounds these challenges. Combining modalities, such as MRI scans and gene expression profiles, requires sophisticated preprocessing strategies to ensure compatibility and retain meaningful relationships across data types. Missing data, a frequent issue in clinical studies, exacerbates these difficulties. While imputation methods are often necessary to address gaps, they frequently introduce variability or bias, which can skew normalization and downstream analyses. Without tailored and standardized preprocessing frameworks, these variations undermine the reproducibility of AI models. This highlights the urgent need for robust strategies to manage the complexities of biomedical data effectively. However, it can be hard to establish standardized complexity management strategies due to the diverse data types, institutional protocols, and evolving privacy requirements that vary across healthcare systems and research settings.

### Model complexity

Model complexity refers to the architectural sophistication and computational demands of AI models. It includes the structural intricacy of models, such as the number of layers and parameters in deep neural networks or transformers, which increases the risk of overfitting and raises computational costs. This complexity is further influenced by advanced architectural designs, including components like attention mechanisms and residual connections, which are used to capture complex relationships in data. Additionally, regularization and optimization techniques, such as dropout and batch normalization, are employed to control overfitting but can introduce variability, adding to the challenge of training complex models. While models with higher complexity often achieve remarkable performance on challenging tasks, this comes at the cost of reduced reproducibility.

#### Model regularization challenges and reproducibility

Model complexity significantly raises the risk of overfitting, and while regularization techniques aim to address this issue, they often introduce additional variations that reduce reproducibility. For instance, dropout, a widely used regularization method, randomly deactivates neurons during training, resulting in different model configurations in each iteration. Although this stochastic behavior enhances generalization by preventing reliance on specific features, it causes variability in learned parameters, leading to inconsistent outcomes across training runs. Similarly, batch normalization introduces stochastic elements by normalizing activations within mini-batches. This method relies on the random sampling of data batches during training, leading to fluctuations in estimated mean and variance. Although effective in reducing overfitting, these techniques create challenges for reproducibility, especially when combined with highly complex model architectures. To some degree, they also exacerbate the complexity of model training and further hinder consistent, reproducible results.

#### Impacts of high model complexity

As model complexity increases, the likelihood of reduced reproducibility grows due to the greater introduction of randomness. For example, models like DeepMind’s AlphaFold3, which feature intricate and highly parameterized architectures for predicting protein folding, are highly sensitive to initial conditions and training data. Even slight variations in input data or training setups can lead to divergent or even amplified outcomes. While these models excel at solving complex problems, their inherent complexity highlights the trade-off between achieving high performance and maintaining reproducibility. Addressing these challenges requires careful consideration of architectural choices, regularization strategies, and training methodologies, which can be impossible to achieve due to the interplay between high data complexity, model complexity, and learning complexity.

### Learning complexity

Learning complexity encompasses the challenges AI models encounter during the process of extracting patterns from data. These challenges arise from the non-deterministic nature of optimization algorithms, the vastness of the solution space, and computational constraints. For instance, non-deterministic optimization, as seen in algorithms like Stochastic Gradient Descent (SGD), introduces variability through the random sampling of data batches, which can lead to different convergence paths, even though SGD is generally robust to overfitting. As a result, models may settle into different local minima on the error surface, thereby affecting reproducibility. Moreover, the task of searching large solution spaces compounds these challenges, as optimal parameter values may remain elusive among countless possibilities. Additionally, it remains an open question how to select or configure an optimizer that balances overfitting risk and reproducibility when tackling AI learning complexity, especially under complicated deep learning models.

#### Generalization

A key aspect of learning complexity is a model’s ability to generalize to unseen data, which requires balancing pattern learning with avoiding overfitting. Similarly to training, achieving this often demands high-performance hardware, like GPUs or TPUs, which, while enabling faster computations, can introduce variability through differences in parallel processing, floating-point precision, and hardware configurations, leading to inconsistent outcomes across experiments [7]. More importantly, hardware variations can exacerbate the risk of overfitting, particularly when they introduce inconsistencies during training. Variability caused by hardware can contribute to this issue by introducing subtle changes in the optimization process, leading the model to overfit certain patterns in the training data.

### Game-theoretical dilemma

Achieving full reproducibility in AI models is undoubtedly challenging, but certain standards can be imposed to improve reproducibility in biomedical data science. For instance, feature selection and normalization procedures can be customized for each dataset to prevent data leakage, ensuring that test data does not inadvertently influence training data. Researchers can also prioritize deterministic AI models and reduce both model and learning complexities by mitigating randomness in optimization, regularization, and parameter tuning. Hardware acceleration methods can likewise be standardized to minimize variability, collectively fostering a more reproducible research environment.

However, researchers often face a game-theoretical dilemma when navigating the landscape of AI reproducibility. While the collective adoption of reproducibility standards benefits the entire scientific community by promoting verifiable and trustworthy research, individual researchers may perceive these standards as restrictive. Rigorous adherence to reproducibility practices can conflict with personal research objectives, such as maximizing model performance, accelerating project timelines, or achieving groundbreaking innovation. These competing priorities often lead to tension between individual ambitions and the communal goal of reliable scientific progress.

This dilemma is not merely theoretical but has practical implications, reflecting the delicate balance between collective benefits and personal incentives. The core of the issue lies in the conflict between the broader advantages of universally applied reproducibility standards and the immediate gains of pursuing novelty and rapid advancement.

## Discussion and conclusion

Reproducibility in AI, particularly within biomedical data science, is a critical yet complex issue that may not be resolved in the short term despite the urgent demand for reliable and verifiable results. The intricate interplay of data, model, and learning complexities, coupled with computational and ethical constraints, creates significant challenges. Addressing these obstacles will require sustained efforts and the development of innovative frameworks to balance reproducibility with efficiency and practicality.

Reproducibility in AI is essential for ensuring reliable outcomes and ethical applications, especially in critical fields like biomedical research. However, the rigorous standards necessary for reproducibility, such as precise data processing and consistent model evaluation, can impose significant costs in terms of learning efficiency and resource demands. For instance, the use of deterministic models and stringent measures to prevent data leakage bolster reproducibility but may limit the adaptability and speed of learning algorithms, particularly when processing large, complex datasets [8]. These requirements often necessitate additional computational steps and resources, which can decelerate model training and lead to secondary-level performance trade-offs.

### Balancing reproducibility and efficiency

Striking a balance between reproducibility and efficiency is challenging and may not be easily achieved. Tailoring reproducibility protocols to different stages of AI development can help; for instance, flexibility during early experimental phases allows researchers to explore innovative methods, while stricter reproducibility controls can be applied as models near clinical application. Streamlining documentation and incorporating automated checks into reproducibility practices can aid in maintaining efficiency without compromising reliability. However, the trade-offs between reproducibility and efficiency remain poorly quantified, and it is unclear how much efficiency can be sacrificed to prioritize reproducibility without jeopardizing the AI system’s reliability, particularly when considering complexities in data, models, and learning processes.

### Game-theoretical dilemma

Collective adherence to reproducibility standards is essential for advancing the broader scientific community, yet individual researchers often prioritize speed and innovation over reproducibility, exacerbating the issue. This misalignment of incentives hampers the adoption of best practices and robust methodologies, creating barriers to consistent reproducibility and to achieving ethical AI in biomedical fields.

Game theory suggests that collective cooperation offers the optimal path forward; however, individual ambitions frequently skew priorities toward short-term gains. Addressing this requires exploring innovative frameworks from a game-theoretical perspective to align researchers’ personal objectives with communal reproducibility goals. Without effectively resolving this conflict, achieving reproducibility in AI will remain a significant challenge, potentially slowing progress in biomedical data science.
